# A Mixed-Methods Study on the Acceptability of Using eHealth for HIV Prevention and Sexual Health Care Among Men Who Have Sex With Men in China

**DOI:** 10.2196/jmir.3370

**Published:** 2015-04-21

**Authors:** Kathryn E Muessig, Cedric H Bien, Chongyi Wei, Elaine J Lo, Min Yang, Joseph D Tucker, Ligang Yang, Gang Meng, Lisa B Hightow-Weidman

**Affiliations:** ^1^Department of Health BehaviorGillings School of Global Public HealthThe University of North Carolina at Chapel HillChapel Hill, NCUnited States; ^2^Icahan School of Medicine at Mount SinaiNew York, NYUnited States; ^3^Department of Epidemiology and BiostatisticsSchool of MedicineUniversity of California, San FranciscoSan Francisco, CAUnited States; ^4^Department of Health Policy and ManagementGillings School of Global Public HealthThe University of North Carolina at Chapel HillChapel Hill, NCUnited States; ^5^Division of Infectious DiseasesSchool of MedicineThe University of North Carolina at Chapel HillChapel Hill, NCUnited States; ^6^Guangdong Provincial Center for Skin Diseases and STI Control & PreventionGuangzhouChina; ^7^Guangtong – Lingnan MSM Community Support CenterGuangzhouChina

**Keywords:** Internet, HIV, AIDS, China, men who have sex with men, mixed method, sexually transmitted diseases

## Abstract

**Background:**

Human immunodeficiency virus (HIV) infection disproportionately affects men who have sex with men (MSM). Over half of all HIV-positive MSM in China may not know their HIV status. Mobile phones and Web interventions (eHealth) are underutilized resources that show promise for supporting HIV education, testing, and linkage to care.

**Objective:**

This mixed-methods study among MSM in China assessed technology utilization and eHealth acceptability for sexual health care.

**Methods:**

We conducted in-depth interviews and an online survey. Qualitative analyses informed the development of the Internet survey, which was administered through two popular MSM websites. Bivariate and multivariate analysis assessed characteristics of MSM interested in eHealth for sexual health care.

**Results:**

The qualitative sample included MSM across a range of ages, education, marital status, sexuality, and HIV testing experience. Qualitative findings included the importance of the Internet as the primary source of information about sexual health, HIV and other sexually transmitted diseases (STDs), use of the Internet to enable HIV testing opportunities by facilitating connections with both the gay community and health care providers, and mixed perceptions regarding the confidentiality of eHealth tools for sexual health. Among the Internet sample (N=1342), the average age was 30.6 years old, 82.81% (1098/1342) were single, and 53.42% (711/1331) had completed college. In the past 3 months, 38.66% (382/988) had condomless sex and 60.53% (805/1330) self-reported having ever tested for HIV. The majority of men owned computers (94.14%, 1220/1296) and mobile phones (92.32%, 1239/1342), which many had used to search for HIV/STD information and testing sites. In multivariate analysis, interest in using computers or mobile phones to support their sexual health care was associated with being a student, prior use of computers or mobile phones to search for general health information, prior use of computers or mobile phones to search for HIV/STD information, and confidentiality concerns.

**Conclusions:**

MSM in this sample had high utilization of technology and interest in eHealth despite confidentiality concerns. Future eHealth interventions can thoughtfully and creatively address these concerns as a priority for successful implementation.

## Introduction

Many men with human immunodeficiency virus (HIV) and who have sex with men (MSM) do not know their HIV status—ranging from around 21% in the United States [1] and New Zealand [2], to over 90% in Malawi [3]. In 2012, China’s national HIV prevalence among MSM reached 6.7% but exceeded 15% in some urban areas [4]. A high national syphilis prevalence around 12% among MSM [5], combined with entrenched fear, stigma, and misunderstanding surrounding HIV and same-sex behavior, inhibit intervention efforts and fuel continued transmission. While government efforts have increased voluntary counseling and testing [6], in 2011 only half of MSM had received an HIV test in the past year and knew their results and as many as 86% of HIV-positive MSM were unaware of their infection [7]. After receiving a positive diagnosis, MSM face delays in care as they pursue required confirmatory and CD4 cell count testing, which are often available only in locations different from the place of original testing and eventual treatment. In this decentralized system, less than half of MSM receive a CD4 count within 6 months of diagnosis [8,9] and under a quarter who qualify for antiretroviral therapy (ART) initiate timely treatment [9]. Delayed linkage to care and suboptimal retention in care hinder effective ART, threaten individual patient outcomes, and may increase the likelihood of onward HIV transmission [10-16].

The tools provided by widespread Internet access and mobile phone ownership offer a promising approach to reducing these gaps in HIV prevention, testing, and care. eHealth approaches support health care services via a wide range of information and communication technologies including software and hardware, Internet and mobile phone-based resources, and global positioning system (GPS) and tablet technologies. Internet and mobile phone-based components have been successfully used within prevention and care initiatives for HIV and other sexually transmitted diseases (STD) [17-19] including promoting prevention messages [20], facilitating test result notification [21,22], and improving ART adherence and attendance at clinic appointments [23-28]. To date, phone-based HIV interventions have primarily utilized voice or text messaging (SMS) functions [19,25,29,30], but smartphones may further facilitate the delivery of more complex, interactive, and tailored interventions via the mobile Web [31,32] and software apps [33,34]. Smartphones facilitate affordable, widespread access to the Internet, narrowing the “digital divide” such that, by 2011, 68% of Chinese mobile phone owners had smartphones [35].

MSM-tailored eHealth initiatives are gaining traction in the United States [31,32,34] but have yet to be systematically designed and tested internationally. The hidden nature of MSM in Chinese mainstream society alongside widespread ownership of mobile technology and the growth of sex seeking websites and online dating services [36,37] make mobile technologies an appropriate, familiar medium to address sexual risk and health promotion. Preliminary survey research suggests that MSM in China are willing to provide Internet and mobile phone personal contact information [38]. In order to develop eHealth interventions specifically for Chinese MSM, additional information is needed about Internet and mobile phone ownership, usage, and preferences for sexual health-related services. Building on eHealth lessons learned in development research with at-risk MSM in the United States [31,32], this mixed-methods study explored how Chinese MSM are currently using the Web and mobile Web to find health information and specifically information related to sexual health. We also aimed to characterize men who are currently willing to use eHealth for sexual health care and to identify possible concerns and barriers to using eHealth. The ultimate goal for these research findings is to inform the design of eHealth interventions to address HIV prevention and sexual health care among MSM in China.

## Methods

### Part I: Qualitative Research

From May to August 2012, we conducted qualitative interviews in two large cities in South China—an area that bears a disproportionate burden of HIV, syphilis, and other STDs especially among MSM [39-43]. The data presented in this manuscript are part of a larger project on MSM testing preferences and experiences. We recruited individuals 16 years of age and older who reported being born biologically male and ever having sex with another man. Participants were recruited through government-sponsored health clinics, non-governmental and community-based organizations, and online through a popular MSM website. We purposively [44] enrolled men across a range of ages, education, marital status, sexuality, and HIV/STD testing experience (never tested, first time being tested, tested multiple times).

Qualitative interviews included questions on sexual health care and HIV/syphilis testing experiences and preferences. Technology questions addressed mobile phone ownership and capabilities, Internet and mobile phone use, experiences and preferences for using technology for general- and HIV/STD-related health care. The interview guide was pre-tested in mock interviews with volunteers at MSM community-based testing centers and subsequently refined. Eligible participants were interviewed one-on-one by trained, bilingual study staff in private rooms at participating study sites. Interviews were conducted in Mandarin, Cantonese, or English, and recorded with permission. Mobile phone cards and supermarket gift cards worth approximately US $10 were offered as remuneration.

All qualitative interviews were transcribed verbatim. The analysis team (KEM, CHB, EJL, JDT) developed a structured codebook to help define and illustrate a priori and emergent themes. Two study team members (CHB, EJL) were trained in how to use the codebook and then independently coded all interview transcripts using Atlas.ti, version 7.0. These coded transcripts were merged and reviewed by a third coder (KEM) for fidelity to the codebook and consistency between coders. No major differences in code applications were identified. All relevant coded data were then reviewed theme by theme and relationships between themes were described. Illustrative quotes were chosen by group consensus to present typical responses and variation within each theme. The findings are organized into four sections as follows: qualitative sample characteristics, the Internet as a primary source of information about sexual health and HIV/STDs, the role of the Internet in facilitating HIV/STD testing, and concerns and considerations for using eHealth for sexual health care among MSM in China.

### Part II: Internet Survey

The qualitative findings informed the design of an Internet-based survey. Survey development was supported by sociologists who conduct the Chinese national survey of sexual behavior [45,46]. The questions were reviewed by four local MSM community volunteers and one focus group discussion. The online survey administration system was pilot tested among 201 MSM (data not included in final analysis). The final survey tool contained 225 possible questions and took an average of 20 minutes to complete. In addition to sociodemographic information, the survey content covered six broad topics: (1) condom and lubrication preferences (results not shown), (2) drug and alcohol use (results not shown), (3) sexual behavior, (4) HIV/STD testing, (5) technology ownership/use, and (6) interest in using eHealth tools for sexual health and confidentiality concerns of using eHealth for sexual health.

In May 2013, the Internet survey was hosted by two high-traffic Web portals: GZTZ.org (Guangzhou) and manbf.com (Chongqing, now manbf.net). These sites were chosen for their hosts’ geographic diversity and widespread popularity among MSM across China. Both websites are run by community-based, MSM-focused organizations founded in 1998 and have social networking and microblogging features. They also advertise MSM-geared products and events, and provide information and services to promote HIV/STD education and testing. Both organizations are community partners with the local Chinese Centers for Disease Control and have received support from international donors including the Global Fund to Fight AIDS, Tuberculosis and Malaria. Guangzhou tongzhi (GZTZ), also known as the Lingnan Group, has been a partner of the China-Gates Foundation HIV Prevention Cooperation Program since 2008. In 2010, GZTZ.org recorded over 3.5 million unique visitors. An assessment of GZTZ users in 2010 found that among 1100 participants, the average age was 30 years old, 93% lived in Guangdong Province, 77.9% self-identified as gay, and 80% had attained a college degree [47]. Manbf.net is the website for the Chongqing Tongxin Working Group and had over 10,000 registered users in 2011.

Banner ads were posted on the Web portal home pages (see Multimedia Appendices 1 and 2). Participants who clicked on the banner ad were taken to a welcome page describing the survey. Those who chose to continue completed an informed consent screener. Consented participants were asked three eligibility questions: born biologically male, at least 16 years old (age of consent in China), and had ever had anal sex with men.

To complete the survey via GZTZ.org, participants had to register and log in. A list of user names was kept (unaffiliated with survey responses) to deter men from completing the survey more than once. In keeping with GZTZ’s past survey research, survey completers were awarded 500 website credits and 50 site “loyalty points” that can be used to “unlock” additional social media features of the GZTZ website. Following the standard practice of the Chongqing Tongxin Working Group at the time, participants recruited through the manbf website were not required to log in and did not receive incentive points.

Frequencies (Table 1) were conducted to describe sociodemographic characteristics, HIV/STD sexual transmission behaviors, and use of computers and mobile phones. To measure interest in using technology as part of their sexual health care, participants were asked to respond “very interested”, “somewhat interested”, or “not interested” for each of the following six items: receiving HIV testing reminders by SMS/text message, receiving HIV testing reminders by QQ (the most popular Chinese chat and instant messaging service) or email, sexual health counseling by online chat, receiving sexual health education via websites, receiving sexual health education via mobile phone apps, and receiving sexual health education via Internet/mobile phone games. Participants who reported being “very interested” in one or more of these six questions were categorized as having “interest in eHealth”, and this variable was used as the main outcome of interest. This decision was made in order to better understand the most likely users for targeting future eHealth interventions.

Univariate and multivariate associations were calculated using logistic regression. A backward elimination process was used to build multivariate models from the variables listed in the tables, with a *P* value of <.10 required for retention in the final model. We assessed collinearity using the variance inflation factor. All statistical analyses were conducted using SPSS, version 21.

The Institutional Review Boards of the Guangdong Provincial STD Control Center, the London School of Hygiene and Tropical Medicine, and the University of North Carolina at Chapel Hill approved this study. See Multimedia Appendix 3 for the Checklist for Reporting Results of Internet E-Surveys (CHERRIES).

**Table 1:** Demographic characteristics of a qualitative and Internet sample of MSM in China.

Demographic and behavioral characteristics		Interviews (N=35), n	Internet survey (N=1342^a^), n (%)
**Recruitment site** ^b^			
	Guangzhou	28	1204 (89.72)
	Hong Kong	7	–
	Chongqing	–	138 (10.28)
**Residence at time of survey**			
	Guangzhou City		799 (59.54)
	Guangdong Province^c^		331 (24.66)
	Chongqing City		124 (9.24)
	Other provinces and Hong Kong^d^		66 (4.92)
	Country outside of China/Hong Kong		15 (1.12)
	Not reported		7 (0.52)
**Official registration permit (hukou)**			
	Guangzhou City		409 (30.48)
	Guangdong Province^c^		485 (36.14)
	Chongqing City		119 (8.87)
	Other provinces and Hong Kong^d^		309 (23.03)
	Country outside of China/Hong Kong		13 (0.97)
	Not reported		7 (0.52)
**Age, mean 30.6, median 29, range 16-64 (n=1327)**			
	16-20	3	37 (2.79)
	21-30	21	733 (55.24)
	31-40	9	440 (33.16)
	>40	2	117 (8.82)
**Marital status (n=1326)**			
	Married or engaged	5	228 (17.19)
	Single	29	1098 (82.81)
**Education (n=1331)**			
	High school or below	7	202 (15.18)
	Some college^e^	14	418 (31.40)
	College or above	14	711 (53.42)
**Living status (n=1326)**			
	Lives alone	–	466 (35.14)
	Lives with others	–	860 (64.86)
**Current employment**			
	Full time	24	1063 (79.21)
	Part time/Retired/unemployed	4	151 (11.25)
	Student	7	128 (9.54)
**Annual income, all sources (USD) (n=1337)**			
	<6000	–	374 (27.97)
	6000-16,000	–	684 (51.16)
	>16,000	–	279 (20.87)
**Sexual orientation (n=1333)**			
	Gay	28	974 (73.07)
	Bisexual	4	352 (26.41)
	Heterosexual	–	7 (0.53)
**Current stable sex partner (n=1327)**			
	Male		590 (44.46)
	Female	–	70 (5.28)
	No stable sex partner	–	33 (2.49)
**HIV testing, lifetime (n=1330)**			
	Never tested	7	525 (39.47)
	Ever tested	28	805 (60.53)
**HIV status**			
	Positive		49 (3.70)
	Negative/unknown		1293 (96.30)
Any condomless anal sex with men, past 3 months		–	382/988 (38.66)
Ever had sex with women (vaginal or anal)		–	448/1333 (33.61)
Any condomless anal sex with women, past 3 months		–	159/1333 (11.93)

^a^Sample is 1342 unless otherwise noted.

^b^Interview recruitment: on site in person. Survey recruitment: 2 websites hosted in Guangzhou and Chongqing, but open to participants from any location.

^c^Cities outside of Guangzhou but within Guangdong Province.

^d^Cities outside of Chongqing and Guangdong Province.

^e^Includes current students.

### Results

### Qualitative Sample Characteristics

#### Overview

Qualitative interviews were conducted with 35 MSM aged 16-48 who were living in Guangzhou and Hong Kong at the time of study recruitment, but whose hometowns represented nine Chinese provinces and Hong Kong. Seven men had never tested for HIV, 7 men had tested for the first time on the day of their interview, and 21 men had tested multiple times. Twenty-eight men self-identified as gay, 4 as bisexual, and 3 chose not to specify. Most participants were unmarried, currently working, and had at least some education beyond high school.

#### The Internet as a Primary Source of Information About Sexual Health and HIV/STDs

Technology ownership and use was widespread in both the qualitative and Internet survey samples. In the qualitative sample, all men owned a mobile phone, the majority of which were smart phones (27/35). Overall, men had a positive response for using phones and the Internet for sexual health. The most common theme was the level of convenience provided for finding information and getting tested. During the interviews, all men reported using mobile phones or computers to search for general health information, and just over half of the men described examples of finding HIV/STD information online for themselves or for friends through community organizations’ websites and other sources:

MSM community organizations’ websites are very important, they contain comprehensive information…about symptoms, characteristics, the window period etc. When my friend was infected with gonorrhea, I actually went online to look up information for him.

B04, gay, multi-time tester, age 27

Notably, the Internet was the primary source of HIV/STD information for many men:

R: Did you know much about HIV or syphilis testing before you looked it up online?

I: No. I searched all the information online first…lots of the knowledge I’ve known until now was from the Internet…Like how to protect yourself. What tools that you can use to protect you and your partner while you are having sex. And HIV information, like today, I searched for organizations to have tests.

A02, gay, multi-time tester, age 24

In the qualitative interviews, 15 men explicitly remembered using their mobile phones to look up sexual health information via mobile search engines (Baidu, Yahoo, Google, Weibo) and blogs.

#### The Internet Facilitated HIV/STD Testing

The Internet facilitated HIV/STD testing by providing information about testing and assistance finding testing facilities; creating safe spaces for men to share testing experiences and information about doctors; exposing men to direct HIV/STD test advertising from community organizations, hospitals and health departments; and providing online appointment scheduling and reminders. For many men, learning about HIV/STD testing happened as they were trying to learn about being gay or connecting with the tongzhi (gay men) community. As men learned about and connected with the tongzhi community online, they were also exposed to HIV/STD information, stories, advertisements, and services targeting MSM:

On the Internet I learned tongzhi is a population with high risk. I got the [testing center’s] address on its website and called to make an appointment…It was a service organization particularly for tongzhi. I am afraid of the way people would look at me if I go to the normal hospital.

A18, gay, multi-time tester, age 41

The Internet also provided opportunities to exchange testing experiences and information about doctors. Men used online recommendations, stories, and reviews to make decisions about testing: “Each place offering testing has its own website. I will look at users’ comments. If the majority of them agree that a place is good, then that would be my choice” (A17, bisexual, first time tester, age 23).

Men who were connected to the online tongzhi community knew that there were service organizations that would provide free, MSM-tailored testing services and described how they used the Internet as a resource to find these services:

If I had a symptom, let’s say persistent low fever, then it’s possible that I was [HIV] positive, and I’d need to have a test. Instead of going to private doctors where I have to pay, I would probably first go online and look up organizations that provide testing for the gay community. Because they are providing the rapid test for free and have better privacy.

B07, gay, never tested, age 35

From a practical standpoint, men used their phones and computers to make testing appointments online, get appointment reminders by text message/SMS, and receive test results by phone call, SMS, or secure website: “Today I used my mobile phone to make my appointment…quite a good experience. Our community organization would inform us when we make an appointment. We would receive text reminders before the appointment telling us the time and duration” (A09, gay, multi-time tester, age 35).

#### Concerns and Considerations

In spite of widespread use and interest in eHealth for sexual health, men also expressed concerns, most commonly around privacy and confidentiality in the need to protect HIV/STD health information from one’s direct social contacts (ie, family, friends, and coworkers) and the general public and government. At the level of privacy from family, friends, and coworkers, the primary concern was that others would directly see HIV/STD information on participants’ phones or computers. This concern also applied to the use of HIV/STD-related mobile phone apps: “I would be a little bit worried about [this information] being on my mobile phone. After all, I’m afraid that somebody will see, and worry about leaks of privacy” (A26, bisexual, multi-time tester, age 48) and “I’ve actually seen some apps [for HIV]…I find them interesting but I will not download them…I sometimes lend my phone to friends for fun. Therefore I would worry they would see those apps” (B01, gay, multi-time tester, age 23).

Participants who already used their computers and phones to search for sexual health information described the protective measures they took including clearing search history, deleting messages, and using phone and Internet passwords. A further recommendation was that future services should use discrete language: “Some people might fear that the messages would be seen by others. That language… could be organized in a way that protects the information, so that other people can’t tell what it’s about” (A20, bisexual, first time tester, age 21).

Men were also concerned about protecting their HIV/STD health information from the general public and the government. In these conversations, phones could help or hinder privacy:

There isn’t much to say about the confidentiality of text messages in China. As you know, all your emails and phone calls can be read or heard by others.

A12, gay, multi-time tester, age 28

Actually, it’s the security of hospital data worth worrying about. In mainland China we have done a poor job of keeping the CDC and hospital data private. Such institutions call patients’ names when it’s ones’ turn to get tested – it’s a big risk for leaking the name of infected patients to the public. Even if the information is not leaked further, it’s still embarrassing. So, the mobile phone security does a better job since only the CDC staff can phone you.

A07, gay, multi-time tester, age 33

Beyond privacy protection, many men also worried about the trustworthiness of online information: “I believe 60% of the websites, which means I suspect 40%. I think a formal website has higher credibility” (A04, bisexual, multi-time tester, age 21). As this man describes, formal sites were perceived as more trustworthy, including familiar MSM organizations and government-supported organizations: “You can search online at China Red Cross and you can search some very official sites like the Guangdong Provincial Health Department. If it’s got ‘Province’ or ‘City’ in the title then it’s official, it can be trusted” (A02, gay, multi-time tester, age 24).

### Internet Survey Sample

For the Internet survey, 3378 participants clicked on the initial link to read about the survey. Among these, 60.49% (2044/3378) consented to participate in the survey. Within these 2044, 6.60% (135/2044) were not eligible due to being born female (5.2%, 7/135), being under the age of 16 (8.1%, 11/135), or reporting never having had sex with a man (86.7%, 117/135). Of the remaining 1909 eligible participants, 1342 (70.30%) finished the survey.

At the time of taking the survey, 59.54% (799/1342) of participants were living in Guangzhou City and 9.24% (124/1342) were living in Chongqing City. Regarding registered residence permit status at the time of the survey: 30.48% (409/1342) were registered in Guangzhou City and 8.87% (119/1342) were registered in Chongqing City (Table 1). For the Internet sample overall, average age was 30.6 years old (range 16-64). Over half of the sample was between 21 and 30 years old. Although the majority of men were single, 64.86% (860/1326) reported living with others (roommates, girlfriend, boyfriend, coworkers, family). Most men had completed an education of college or beyond, and 9.54% (128/1342) were currently full-time students. About three quarters of the sample self-identified as tongzhi (gay). Annual income varied with 27.97% (374/1337) earning less than US $6000 per year. Over one-third of men had never tested for HIV. Among those who had tested and knew their results, 6.1% (49/805) were HIV-positive. A substantial proportion of men reported having condomlesss sex with men (38.7%, 382/988) or women (11.93%, 159/1333) in the past 3 months (Table 1) including 39% (19/49) of men who reported being HIV-positive.

All of our qualitative findings were confirmed in the Internet survey (Table 2): 92.32% (1239/1342) of survey respondents owned a mobile phone or a smartphone and 94.14% (120/1296) owned a computer. The majority (88.05%, 1009/1146) of men connected to the Internet at least once a day and 83.71% (956/1142) used mobile phone apps at least once a day. In the online survey, men reported using computers and mobile phones to search online for general health information (computer: 63.65%, 746/1172; mobile phone: 41.48%, 465/1121) as well as specific information about HIV/STDs (computer: 51.80%, 603/1164; mobile phone: 27.64%, 306/1107).

Most men expressed interest in using eHealth modalities for sexual health (Table 2), including receiving HIV testing reminders by SMS/text message, or QQ chat/email; sexual health counseling by online chat; and sexual education via websites, mobile phone apps, or Internet/mobile phone games. Overall 43.89% (589/1342) of men were very interested in at least one of these eHealth modalities (main outcome), and 29.06% (390/1342) were somewhat or very interested in all six items.

**Table 2:** Computer and mobile phone usage and acceptability of technology-based sexual eHealth intervention among 1342 MSM in China (N=1342^a^).

Characteristics		n (%)
**Technology ownership**		
	Mobile phone	1239/1342 (92.32)
	Smartphone	1146/1225 (93.55)
	Computer	1220/1296 (94.14)
**Phone service carrier (n=1216)**		
	CMCC	773 (63.57)
	China Unicom	273 (22.45)
	China Telecom	149 (12.25)
	Other	21 (1.73)
**Years using current phone number (n=1226)**		
	Less than 3 years	514 (41.92)
	3 or more years	712 (58.08)
**Main study outcome: Very interested in one or more of the following**		
	Receiving HIV testing reminders by SMS or text message	247/1159 (21.31)
	Receiving HIV testing reminders by QQ chat or email	241/1139 (21.16)
	Sexual health counseling by online chat	335/1129 (29.67)
	Sexual health education via websites	386/1142 (33.80)
	Sexual health education via mobile phone apps	306/1126 (27.18)
	Sexual health education via Internet/mobile phone games	262/1106 (23.69)
Use mobile phone apps daily		956/1142 (83.71)
Use Internet daily		1009/1146 (88.05)
**Past eHealth use, HIV/STD**		
	Used mobile phone to find HIV/STD testing site	298/1165 (25.58)
	Used mobile phone to find other information about HIV/STD	306/1107 (27.64)
	Used computer to find HIV/STD testing site	601/1227 (48.98)
	Used computer to find other information about HIV/STD	603/1164 (51.80)
**Past eHealth use, general**		
	Used mobile phone for general health information	465/1121 (41.48)
	Used computer for general health information	746/1172 (63.65)
**Any confidentiality concerns**		
	Concerns receiving HIV/STD-related SMS or text message	962/1170 (82.22)
	Concerns receiving HIV/STD-related QQ chat or email message	946/1163 (81.34)

^a^The total sample size was 1342. Due to programmed skip patterns and participant choice to refrain from answering some questions, denominators in this table can vary from 1342. For example, all participants responded to the question “Do you own a mobile phone?” (1239/1342). The 1239 participants who responded “yes, I own a mobile phone” were then asked, “Is your mobile phone a smartphone?” Among the 1225 participants who chose to answer this question, 93.55% (1146/1225) reported that their mobile phone is a smartphone.

In bivariate analysis (Table 3), being very interested in using computers or mobile phones for sexual health was positively associated with student status, lower annual income, past HIV testing, being self-reported HIV-positive, daily mobile phone app use, daily Internet use, past use of computers or mobile phones to find HIV/STD information or testing sites, past use of computers or mobile phones to find general health information, and confidentiality concerns about using eHealth for sexual health.

**Table 3:** Correlates of interest in technology for sexual health among 1342 MSM in China.

Characteristics		Very interested in using technology for sexual health (n=589, 43.89%)					
		n/N (%)	OR (95% CI)	*P*	Adjusted OR (95% CI)		*P*
**Age**							
	Over 40	45/117 (38.46)	1				
	31-40	186/440 (42.27)	1.17 (0.77-1.78)	.46			
	21-30	333/733 (45.43)	1.33 (0.89-1.99)	.16			
	16-20	19/37 (51.35)	1.69 (0.80-3.56)	.17			
**Education**							
	College or above	304/711 (42.76)	1				
	Some college	183/418 (43.78)	1.04 (0.82-1.33)	.74			
	High school or less	97/202 (48.02)	1.24 (0.90-1.69)	.18			
**Annual income, RMB**							
	˃16000	124/279 (44.44)	1		1		
	6000-16,000 RMB	270/684 (39.47)	0.82 (0.62-1.08)	.16	0.76 (0.54-1.05)		.09
	˂6000 RMB	194/374 (51.87)	1.35 (0.99-1.84)	.06	1.01 (0.67-1.52)		.96
**Sexual orientation**							
	Bisexual/heterosexual/other	151/359 (42.06)	1				
	Gay	437/974 (44.87)	1.12 (0.88-1.43)	.36			
**Living status**							
	Lives alone	193/466 (41.42)	1				
	Lives with others	390/860 (45.35)	1.17 (0.93-1.47)	.17			
**Marital status (to women)**							
	Single/divorced/widowed	489/1098 (44.54)	1				
	Married/engaged	93/228 (40.79)	0.86 (0.64-1.15)	.30			
**Employment**							
	Unemployed/part-time/ retired	57/151 (37.75)	1		1		
	Full-time employed	451/1063 (42.43)	1.22 (0.86-1.73)	.28	1.20 (0.76-1.90)		.43
	Student	81/128 (63.28)	2.84 (1.75-4.63)	<.001^a^	2.27 (1.24-4.16)		.008^a^
**Study site**							
	Guangzhou	522/1204 (43.36)	1				
	Chongqing	67/138 (48.55)	1.23 (0.87-1.75)	.25			
**Migrant status**							
	Guangdong or Sichuan	440/994 (44.27)	1				
	Other	145/332 (43.67)	0.98 (0.76-1.25)	.85			
**Has a current stable sex partner**							
	No	276/634 (43.53)	1				
	Yes	312/702 (44.44)	1.04 (0.84-1.29)	.74			
**Unprotected anal intercourse in past 3 months (men/women)**							
	No	427/971 (43.98)	1				
	Yes	134/306 (43.79)	0.99 (0.77-1.29)	.96			
**Ever tested for HIV**							
	No	193/527 (36.62)	1		1		
	Yes	396/815 (48.59)	1.64 (1.31-2.05)	<.001^a^	1.15 (0.86-1.52)		.35
**HIV status**							
	Negative/unknown	560/1293 (43.31)	1		1		
	HIV-positive	29/49 (59.18)	1.90 (1.06-3.39)	.03^b^	1.33 (0.64-2.74)		.44
**Use mobile phone apps daily**							
	No	72/186 (38.71)	1		1		
	Yes	452/956 (47.28)	1.42 (1.03-1.96)	.03^b^	1.29 (0.84-1.98)		.25
**Use Internet daily**							
	No	118/333 (35.44)	1		1		
	Yes	471/1009 (46.68)	1.60 (1.23-2.06)	<.001^a^	1.08 (0.67-1.74)		.76
**Past eHealth use, HIV/STD** ^c^							
	No	174/627 (27.75)	1		1		
	Yes	415/715 (58.04)	3.60 (2.86-4.53)	<.001^a^	2.11 (1.53-2.90)	<.001^a^	
**Past eHealth use, general health**							
	No	153/561 (27.27)	1		1		
	Yes	436/781 (55.83)	3.37 (2.67-4.26)	<.001^a^	1.70 (1.23-2.33)		.001^a^
**Any confidentiality concerns**							
	No	46/302 (15.23)	1		1		
	Yes	543/1040 (52.21)	6.10 (4.35-8.55)	<.001^a^	3.70 (2.47-5.54)	<.001^a^	

^a^Significant at *P*<.01.

^b^Significant at *P*<.05.

^c^“Past eHealth use for HIV/STI” is a combined variable based on four variables in Table 2 (any past reported use of mobile phone or computer to search for HIV/STI testing site or other information about HIV/STI).

In multivariate analysis (Table 3), four variables remained significantly associated with interest in eHealth: student status (adjusted OR 2.27, 95% CI 1.24-4.16, *P*=.008), past use of eHealth (mobile phone or computer) to search for HIV/STD information or testing site (adjusted OR 2.11, 95% CI, 1.53-2.90, *P*<.001), past use of eHealth to search for general health information (other than HIV/STDs) (adjusted OR 1.70, 95% CI 1.23-2.33, *P*=.001), and having any confidentiality concerns related to eHealth (adjusted OR 3.70, 95% CI 2.47-5.54, *P*<.001).

## Discussion

### Principal Findings

The purpose of this mixed-methods study among a total of 1377 MSM across multiple sites in China was to conduct formative work to inform the development of future eHealth interventions. We found high ownership, utilization, and interest in computer and mobile phone–based tools to support sexual health care, showing promise for the expansion of eHealth HIV/STD interventions among MSM in China. In this Internet-based sample, MSM who expressed interest in the use of eHealth as part of their sexual health care were more likely to have previously used mobile phones or computers to find HIV/STD information but also had confidentiality concerns about eHealth. Evidence from our qualitative sample elucidated ways men are already using these tools and how they facilitated connections to HIV/STD testing and health services. eHealth tools address barriers to better HIV/STD prevention, linkage to care, and treatment. In particular, men in our sample described how the tools of eHealth addressed barriers to prevention and care activities for them. For example, the Internet provided a way to find testing sites and schedule appointments (removing logistical barriers), identify free services (removing financial barriers), and reduce fear of testing via reading about other men’s testing experiences (removing psychosocial barriers).

Our findings speak directly to a recent study among 404 MSM in China that found a lack of HIV testing was associated with not knowing where to get tested, limited HIV knowledge, low perceived HIV risk, concerns about the confidentiality of HIV testing, not being openly gay, not using the Internet, and not having others who had tested for HIV in their social network [48]. Our qualitative interview findings show how Internet use among MSM in China is already addressing each of these barriers and suggest ways for expanding future eHealth interventions. For most men in our qualitative sample, the Internet was an equally, if not more important source of information about HIV/STDs and testing sites than close friends or health professionals. Men gleaned information from formal sites (health departments, hospitals, community-based organizations) as well as blogs and posts of other men’s experiences, reviews, and recommendations. Online advertisements, information, and direct invitations from MSM community-based organizations successfully prompted some MSM to get HIV/STD testing.

Importantly, interactive multimedia has high appeal across gender, culture, and age groups. eHealth modalities are now being used to deliver and address many aspects of HIV prevention including in diverse settings in China such as Web-based HIV/AIDS education and stigma reduction intervention in rural areas [49] and sex education and awareness raising among urban youth [50]. Community-based MSM organizations in China are already trying these technologies. For example, a mobile phone social and sexual networking app called Blued (similar to Jack’d) has been used to promote HIV testing among MSM in Beijing [51]. In addition, the GZTZ organization has created an online program called “Yigaozhi” (Easy to Tell), which helps HIV-positive MSM with status disclosure and HIV test promotion among their sexual partners [52]. The benefits offered by eHealth include broad reach and accessibility to wide audiences; the ability to create interventions that are brief, flexible, and tailored to individual users; and cost savings through scalability, widespread dissemination, and reduced burden compared to face-to-face interventions.

As highlighted in our qualitative interviews, men were aware of the varying quality of information provided online and had developed strategies for coping with this complication including seeking information from sources they perceived to be more “official” such as the Red Cross or provincial-level websites. Credibility is an important consideration in developing eHealth interventions for MSM in China in terms of choosing where and how information will be delivered. There is also room for future research that assesses the accuracy of health information currently being provided by popular MSM websites in China.

Many men accessed the Internet via their mobile phones to look up sexual health information, make appointments, and receive HIV/STD testing information and/or test results. However, overall lower access of the Internet by mobile phone (as compared to computers) in the context of high smartphone ownership indicates an area for development and expansion in optimizing Web and mobile phone apps to make online information more accessible and user-friendly via mobile phones. MSM websites and online services should be visually optimized for mobile phone access to get the broadest use in real-time. Additional tools for future adaptation and development could include websites that include more interactive features, peer and/or health provider facilitated QQ groups and online forums, expansion of reminder and result notification texting/SMS programs, GPS-enabled apps for locating free HIV/STD testing and condoms (eg, NYC Condom, Philly Condom), and mobile phone apps to support HIV care appointments and medication adherence. For example, most men found out about HIV/STD testing online and many men had used online services to schedule appointments. However, there was often a time lag of months or even years between learning about testing and getting tested. This suggests an opportunity for an eHealth-based tool that would facilitate linkage between awareness of testing and actual testing. This kind of tool could include elements such as online personal risk assessment quizzes and automated or personalized follow-up test scheduling prompts and reminders.

To reach the widest population of MSM, eHealth approaches for HIV/STD testing and care should offer a variety of modalities (calls, text/SMS, Web-based resources, online chats/forums, apps, etc) and services. In our Internet survey, MSM preferences for eHealth modalities varied. Sexual health websites, online chat, and mobile phone apps were the most popular. Furthermore, in the qualitative sample, men described a wide variety of ways that they used online HIV/STD information and services. For successful intervention, the mode of eHealth delivery matters. For example, a 2008 randomized controlled trial designed to reduce HIV risk among MSM in Hong Kong failed to achieve significant changes in any of the intervention outcomes [53]. This intervention was primarily delivered to participants by email and followed an information format. Similarly, a multicomponent sexual education Internet-based intervention found that both the email and discussion board features of the intervention were underutilized [50]. In further support of the importance of delivery mode, Zou et al found that MSM in China who were recruited through instant messaging (QQ), online chat, and mobile phone contact (text, SMS) were significantly more likely to actually go and get tested compared to men who were recruited via email [54].

In general, MSM in our study were willing to use their phones to get HIV/STD information and services, but many were concerned about confidentiality and privacy. In the Internet sample, interest in eHealth was strongly associated with confidentiality concerns. This finding may seem counterintuitive; however, it may also signify an unmeasured confounder or construct such as prior negative experiences with breach of confidential health information within the mainstream medical system or general concern about monitoring of the Internet. Further work is needed to better understand the sources of these concerns and how they might be addressed. In the qualitative sample, participants expressed a range of opinions regarding the security of accessing and retaining health information by Internet and on their phones. While some men were concerned about the confidentiality of health information exchanged by phone, others felt that phones were more confidential than paper records or in-person consultations. Those who were using phones and computers for sexual health suggested a range of solutions to protect confidential information including using indirect language, keeping phones locked, and regularly deleting messages and browser history. Overall, men’s confidentiality concerns emphasize the importance of sensitivity to this issue as well as building and clearly communicating security features of future eHealth interventions for MSM in China.

### Limitations

This study is subject to several limitations. Internet- and venue-based samples are subject to different kinds of recruitment bias. While we cannot quantify the extent of these biases, a strength of our study lies in its use of both recruitment modes and sample findings that mutually reinforced each other, increasing the reliability of both samples. Two additional questions of greatest interest that address the validity of using our findings for intervention recommendations are (1) Does Internet-based recruitment of Chinese MSM capture an appropriate at-risk population for HIV/STD intervention? and (2) How representative is our sample of the broader population of Internet-using MSM in China? In our online sample, men reported high levels of condomless sex with men and women, and low levels of HIV testing. This finding reinforces previous research among MSM in China that found high levels of sexual risk behaviors among Internet-based samples [55-57]. Furthermore, evidence of heightened risk of HIV has been demonstrated among MSM in China who use the Internet. Among a sample of 77 recently diagnosed HIV-positive MSM, 58.4% had found sex partners via the Internet during the year leading up to their infection [58], and among 307 young migrant MSM, men recruited via the Internet had higher prevalence of HIV as compared to those recruited from venues, peer outreach, or network referrals [59].

The health education promotion activities on the two survey host websites may also create a source of bias whereby our recruited participants had higher willingness to utilize eHealth interventions (as compared to the general population of Internet-using MSM). While this is possible, our study found that in spite of exposure to these websites, 38.7% of survey respondents reported condomless sex in the past 3 months and 39.5% had never been tested for HIV. These continued risk behaviors among MSM who are utilizing websites that promote health activities suggest that there is room for intensified or adapted eHealth intervention activities targeting this population. Furthermore, our study does not specifically address MSM who do not have access to (or choose not to use) the Internet and/or mobile smartphones. While likely a growing minority in China’s rapidly expanding technology market, these men also need continued, targeted outreach as they are likely to be overrepresented in the lowest socioeconomic strata of MSM in China [5,57].

An additional limitation of our study was the different completion rates between our two recruitment sites. Among screened, eligible participants, 36.5% of Chongqing participants went on to complete the survey versus 78.6% of Guangzhou participants. However, for potential participants who completed the initial screener questions (sex assigned at birth, current self-identified gender, lifetime unprotected anal intercourse, age), we found no significant difference between sites and between those who went on to take the survey versus those who did not. We suspect the difference in the proportion of those who went on to take the survey was most directly related to the lack of incentive provided for the Chongqing participants. One goal in conducting the study was to be least disruptive to the current norms among these well-established online MSM communities. Therefore, we followed each organization’s standard practice for remuneration in keeping with their prior research and site management conventions. Given the minor nature of the incentive provided (no money or physical item directly given), we do not feel that the incentive would have caused a meaningful difference in the characteristics of who completed the survey. In addition, we found no association between recruitment site and our outcome variable. We also conducted a sensitivity analysis including recruitment site in the multivariate model and found no significant differences (data not shown). Nevertheless, the varying survey completion rate is an important finding on its own as future Internet-based surveys that attempt to utilize multiple recruitment platforms will face similar questions of following existing remuneration practices versus imposing other standards. It is also promising to note that even a small, non-monetary incentive can serve as sufficient remuneration for eliciting high online survey participation.

### Conclusions

To date, meta-analyzed results of interventions among MSM in China have shown significant changes in HIV knowledge and sexual risk behaviors but have not achieved the hoped-for level of change in HIV incidence [60]. The field of HIV prevention is shifting to combination approaches with greater consideration of social and structural risk factors as well as incorporation of biomedical advances (eg, point-of-care rapid HIV, CD4, and viral load testing; ART treatment as prevention, pre-exposure prophylaxis), alongside traditional individual-level education and behavior. Our study has implications for the new “test and treat” models that are being promoted as part of these comprehensive HIV intervention strategies [61]. eHealth interventions can capitalize on the social networking and support available through the Internet and can play a key role along each step of the HIV testing and care cascade from connecting participants to testing centers to helping HIV-infected persons with daily medication adherence [62]. For individual-level interventions, formative work—as we have reported here—and pilot testing among the study population are critical steps for identifying the best modes and messages to improve the chances of intervention efficacy and acceptability. Although Internet and mobile phones are ubiquitous in China, their full potential has not yet been optimally employed to support health interventions for HIV/STD prevention, testing, linkage, and care. eHealth interventions could capitalize on widespread mobile phone ownership and build on preliminary work that establishes the acceptability and current use of these technologies to support MSM health and sexual health in particular.

## Multimedia Appendix 1

Survey banner advertisement ("Participate in this survey to quickly earn 50 loyalty points. NEW!").

## Multimedia Appendix 2

Survey banner advertisement ("Survey click here to attend: Understanding MSM's experience and perspectives about testing and obtaining sex products").

## Multimedia Appendix 3

Checklist for Reporting Results of Internet E-Surveys (CHERRIES).

## Abbreviations

AIDS: acquired immune deficiency syndrome
ART: antiretroviral therapy
CD4: cluster of differentiation 4, glycoprotein found on the surface of immune cells
CMCC: China Mobile Communications Corporation
eHealth: electronic Health
GPS: global positioning systems
GZTZ: Guangzhou Tongzhi
HIV: human immunodeficiency virus
MSM: men who have sex with men
QQ: Tencent QQ, a popular instant messaging software service in China
SMS: short message service/text messaging
STD: sexually transmitted disease
STI: sexually transmitted infection
