# Supplementary figures and images for: A Mixed-Methods Study on the Acceptability of Using eHealth for HIV Prevention and Sexual Health Care Among Men Who Have Sex With Men in China
Source: J Med Internet Res. 2015 Apr 21;17(4):e100. doi: 10.2196/jmir.3370 (PMC4420841; doi:10.2196/jmir.3370)

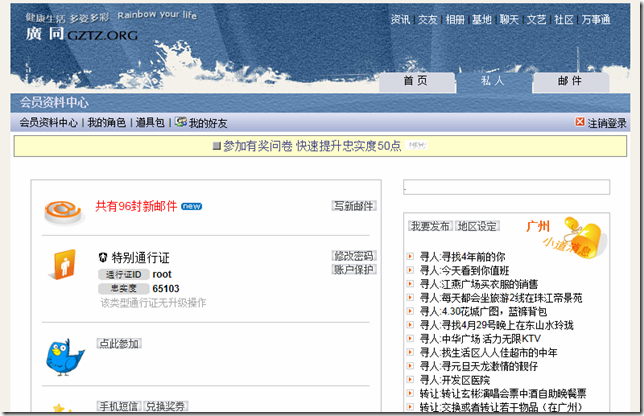

Supplement: Supplementary file 1 [file jmir_v17i4e100_app1.jpg]

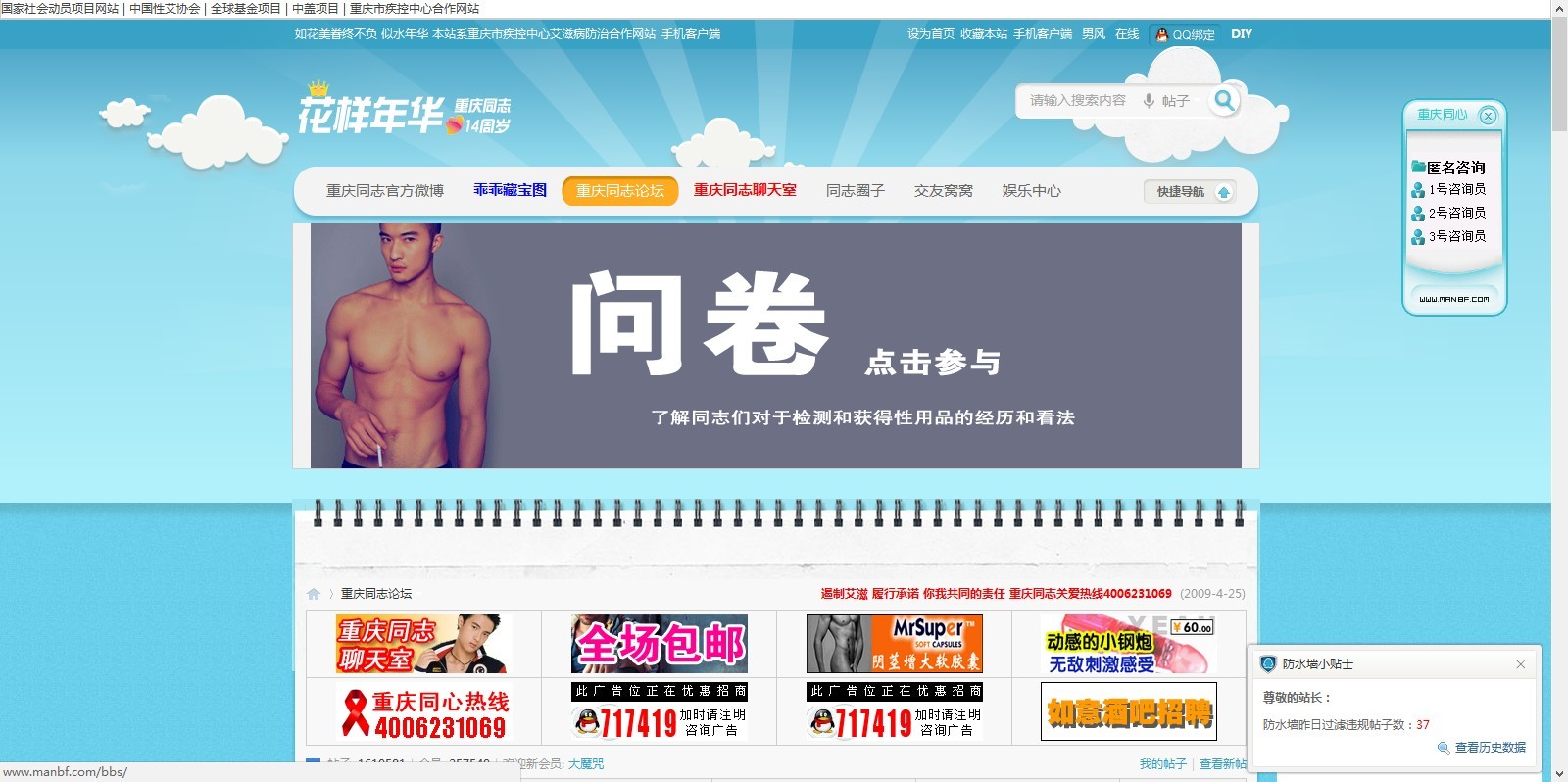

Supplement: Supplementary file 2 [file jmir_v17i4e100_app2.jpg]
