# Supplementary material for: A Mixed-Methods Study on the Acceptability of Using eHealth for HIV Prevention and Sexual Health Care Among Men Who Have Sex With Men in China
Source: J Med Internet Res. 2015 Apr 21;17(4):e100. doi: 10.2196/jmir.3370 (PMC4420841; doi:10.2196/jmir.3370)
Supplement: Supplementary file 3 [file jmir_v17i4e100_app3.pdf]

Supplemental Table 1: Checklist for Reporting Results of Internet E-Surveys (CHERRIES)

Item Category	Checklist Item	Explanation
**Design**	Describe survey design	Target population: Chinese men who reported ever having sex with men. Survey topics: sociodemographic information, condom and lubrication preferences, sexual behavior, drug and alcohol use, HIV/STD testing and treatment, technology ownership/use, and interest in using eHealth tools for sexual health. The survey was designed with extensive formative research and hosted in Chinese by two high-traffic web portals (Guangzhou Tongzhi – GZTZ; and AIDS Care China) which were chosen for their hosts' geographic diversity and widespread popularity among Chinese MSM.
**IRB approval and informed consent process**	IRB approval	IRBs of the Guangdong Provincial STD Control Center, the London School of Hygiene and Tropical Medicine, and the University of North Carolina at Chapel Hill reviewed and approved this study.
	Informed consent	Banner ads were posted on the web portal home pages. Visitors who clicked on the banner ad were taken to a welcome page describing the survey including length of the survey, purpose of the study, investigators, and how data would be collected and stored. Those who chose to continue clicked through to a second screen to review an informed consent page. Consented participants were then asked three eligibility questions: born biologically male, at least 16 years old (age of consent in China); and, had ever had anal sex with men.
	Data protection	No personal identifying information was collected. The survey was hosted and all data stored on a secure server.
**Development and pre-testing**	Development and testing	Qualitative findings from an extensive formative study with Chinese MSM and stakeholders informed the design of the internet survey. Survey development was supported by one survey design specialist at the University of North Carolina and a team of sociologists from the Chinese national survey of sexual behavior. The survey tool was translated into Chinese and back translated into English by bilingual speakers. The questions were

		reviewed by four local Chinese MSM community volunteers and one focus group discussion with MSM. The online survey administration system was pretested and then pilot tested live for 24 hours yielding 201 MSM respondents. This data was not included in final analysis but was analyzed to adjust the survey for any errors.
**Recruitment process and description of the sample having access to the questionnaire**	Open survey versus closed survey	The survey was open to participants who met the three eligibility criteria presented in the pre-survey screener. Eligibility criteria were not made known to participants prior to viewing the questions so as to encourage honest responses. To complete the survey via GZTZ, participants had to register a username with an email address or phone number and log in. Following the standard practice of the AIDS Care China website, participants recruited through this portal were not required to register or log in.
	Contact mode	Banner ads for the study were posted on the web portal home pages. No contact was made with participants outside of the Internet-based survey.
	Advertising the survey	Banner ads were posted on the web portal home pages.<br><br>The translated banner ad announcements stated: "Survey click here to attend: understanding MSM's experience and perspectives about testing and obtaining sex products" or "Participate in this survey to quickly earn 50 loyalty points. NEW!"<br><br>The survey announcements (in Chinese) are published as an appendix.
**Survey administration**	Web/E-mail	The survey was administered via the MSM community centers' websites.
	Context	The survey was hosted by two high-traffic web sites GZTZ and AIDS Care China (Chongqing). These sites were chosen for their hosts' geographic diversity and widespread popularity among MSM. MSM are the primary audiences for these websites. The sites provide HIV/STI and men's health-related information and service information as well as opportunities for discussion forums, advertisements of MSM targeted products, and announcements of news and events.
	Mandatory/voluntary	The survey was completely voluntary. Users could access the websites without completing the survey.

	Incentives	To complete the survey via GZTZ, participants had to register and log in. In keeping with GZTZ's past survey research, survey completers were awarded 500 website credits and 50 site "loyalty points" which can be used to "unlock" additional social media features of the GZTZ website. Following the standard practice of the AIDS Care China website, participants recruited through this portal did not receive incentive points for survey completion.
	Time/Date	The survey was available online from May 1 – 31, 2013.
	Randomization of items or questionnaires	Survey items were not randomized as each subsequent section of the survey intentionally built on the prior section.
	Adaptive questioning	Complex skip patterns and adaptive questioning was programmed into the survey to reduce number and complexity of the questions.
	Number of Items	The final survey tool contained 225 total possible questions and took an average of 20 minutes to complete.<br><br>An average page contained 4-5 questions (range 1-10 questions per page depending on question length)
	Number of screens (pages)	The total number of pages a participant could see was 38.<br><br>Due to the extensive skip patterns, the total number of pages a given respondent would see could be less than this.
	Completeness check	All questions items provided a non-response option (either "not applicable" or "decline to answer") Each question required a response in order to advance to subsequent question screens. A completeness check was conducted after the questionnaire was submitted during the analysis phase.
	Review step	Due to the length of the survey, participants were not required to review their responses at survey completion. A "back" button was provided if participants wished to edit previous answers.
**Response rates**	Unique site visitor	Google analytics was used to calculate unique site visitors with a cookie-based tracking system (https://support.google.com/analytics/answer/2992042?hl=en)
	View rate	Requires counting unique site visitors (not

	(Ratio unique site visitors/unique survey visitors)	page views!) divided by the number of unique visitors of the first page of the survey. It is not unusual to have view rates of less than 0.1% if the survey is voluntary.<br><br>Overall visitors to the main home page hosting the banner ad at GZTZ from May 1 – 31, 2013: 45,157.<br><br>We did not track how many people clicked on the banner ads.
	Participation rate (Ratio unique survey page visitors/agreed to participate)	For the internet survey, 3378 participants completed informed consent, of which 1935 (57.28%) were eligible and initiated the survey.
	Completion rate (Ratio agreed to participate/finished survey)	1342 out of these 1935 (69.35%) finished the survey.
**Preventing multiple entries from the same individual**	Cookies used	Authentication cookies are used by GZTZ website to identify site members at log-in.
	IP check	IP addresses were not collected from participants as we anticipated that many participants would complete the survey via public computers in internet cafes, community organizations, or other multi-user computers.
	Log file analysis	On the GZTZ website, a username was required to gain access to the survey. A list of usernames and assigned study ID numbers was kept (unaffiliated with survey responses) on file. The same username was not permitted to complete the survey more than once on the GZTZ website. Since the study incentive was linked to the specific username account and was minimal, it is unlikely that an individual would be motivated to create a second username in order to complete the survey again. On the AIDS Care China website, no usernames were required to complete the survey, however, no participant incentive was provided, decreasing the likelihood of completing the survey multiple times.

**Analysis**	Handling of incomplete questionnaires	Questionnaires with at least 80% of applicable questions completed were analyzed. Sociodemographic questions were inserted throughout the survey (rather than at the end of the survey) in order to increase capture of this information.
	Questionnaire s submitted with an atypical timestamp	Time stamps were collected at start and completion of the survey. Due to the complexity of skip patterns, the length of time required to complete the survey varied widely across participants, thus no cut-offs were used based on time-stamp measures. Instead, responses for participants with unusually short completion rates (under 10 minutes – chosen based on the most minimal set of questions possible) were examined for inconsistencies in responses that would suggest random “click-through”.
	Statistical correction	No statistical correction procedures or weightings were used in the analysis.

Eysenbach, G. (2004). Improving the quality of web surveys: the checklist for reporting results of internet e-surveys (cherries). *Journal of medical Internet research*, 6(3)e34 doi:10.2196/jmir.6.3.e34. http://www.jmir.org/2004/3/e34/
